# Protective Effects of Polyphenols Present in Mediterranean Diet on Endothelial Dysfunction

**DOI:** 10.1155/2020/2097096

**Published:** 2020-08-06

**Authors:** K. Stromsnes, C. Mas-Bargues, J. Gambini, L. Gimeno-Mallench

**Affiliations:** Freshage Research Group, Department of Physiology, Faculty of Medicine, University of Valencia, CIBERFES, INCLIVA, Avenida Blasco Ibañez, 15 46010 Valencia, Spain

## Abstract

Endothelial dysfunction tends to be the initial indicator in proinflammatory state and macro- and microvascular complications, such as atherosclerosis and cardiovascular diseases. It has been shown that certain compounds in diet can generate beneficial effects on cardiovascular disease due to its interactions with endothelial cells. Thus, this review is aimed at investigating whether certain polyphenols present in the Mediterranean diet, specifically catechin, quercetin, resveratrol, and urolithin, could exert positive effects on endothelial dysfunction. After analysis of numerous papers, we found that polyphenols aiding endothelial function is beneficial not only for patients with cardiovascular disease, diabetes, or endothelial dysfunction but for all people as it can improve the effects of aging on the endothelia. The additional benefit of these polyphenols on weight loss further improves health and lowers the risk of several diseases, including those caused by endothelial dysfunction. However, it is important to note that the dosages in the majorities of the studies mentioned in this review were of supplemental rather than nutritionally relevant quantities, and therefore, the recommended dosages are difficult to determine.

## 1. Introduction

### 1.1. Endothelial Function

Vascular endothelial cells line the entire circulatory system, from the heart to the smallest capillaries, and play a crucial role in maintaining cardiovascular homeostasis, attenuating vascular inflammation and controlling blood flow and vascular tone [1, 2].

**Figure 1:**
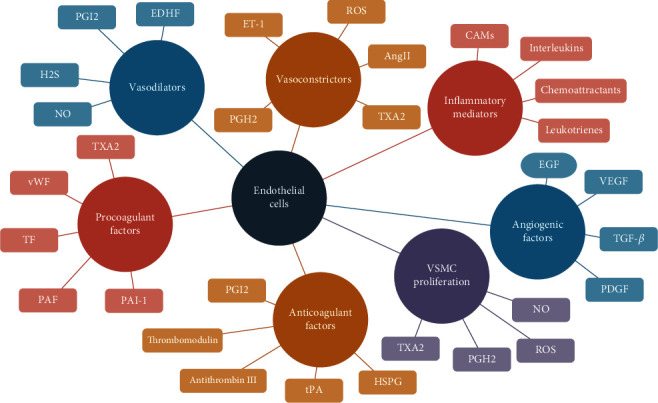
Endothelial function mediators [6], modified from Sena 2018.

A shift in the functions of the endothelium can cause dysfunction through vasoconstriction, proinflammatory and prothrombic states [2, 3]. Endothelial dysfunction tends to be the initial state in macrovascular complications such as coronary artery disease, peripheral arterial disease, and stroke. It may also lead to microvascular complications such as nephropathy, neuropathy, and retinopathy. Numerous strategies have been developed to protect endothelial cells, of which the role of polyphenolic compounds in modulating the differentially regulated pathways has been proven to be beneficial [4]. Control of the endothelial functions is achieved through the production and release of various mediators, such as the vasoconstrictor endothelin-1 (ET-1) and relaxant nitric oxide (NO) that modify the responsiveness of the underlying vascular smooth muscle [3, 5] (see Figure 1) [6].

The figure shows the different local factors that directly affect the function of endothelial cells. Vasodilators and vasoconstrictor modify the caliber of blood vessels. Coagulating and anticoagulating factors permit an optimal control of the hemostasis and impede spontaneous coagulations, which are also influenced by inflammatory mediators. Together with angiogenic factors and proliferation regulation, optimal cell replacement and reparation are achieved. Generally, anti-inflammatory mediators and dilating, proliferation, angiogenic, and procoagulant factors tend to be beneficial for diseases related to the cardiovascular system. However, these should exist in a balanced state with their opposing factor/mediator to ensure correct endothelial function.

### 1.2. Polyphenols

Polyphenols are chemical compounds synthesized by plants containing at least one aromatic ring and a hydroxyl group [7]. They are the main source of antioxidants in diet, as they are abundant in plants, including fruits, nuts, vegetables, and cereals, as well as derived beverages such as tea, coffee, and wine [8]. There are more than 8000 known polyphenols, which are classified into phenolic acids, stilbenes, phenolic alcohols, lignans, and flavonoids [8, 9]. Polyphenols have spiked great interest in the scientific community due to their health benefits, particularly in relation to cardiovascular diseases [10]. These exerted effects are suggested to include anti-inflammatory, antithrombotic, anticoagulant, antimicrobial, vasodilatory, and anticarcinogenic protection [10].

### 1.3. Mediterranean Diet

The Mediterranean diet is widely known for being rich in fruits, vegetables, legumes, nuts, and grains with a moderate intake of red wine and reduced consumptions of meat and dairy. Due to the antioxidant and anti-inflammatory properties of plant foods and the low intake of saturated fats, those following a Mediterranean diet have been shown to have lower incidence of obesity, insulin resistance, endothelial dysfunction, and cardiovascular disease [11, 12].

Red wine is one of the components present in this diet and is thought to be responsible for the “French-paradox”, which describes the low risk of cardiovascular disease despite a diet rich in saturated fats and cholesterol [13]. Red wine contains, among other compounds, quercetin, catechin, resveratrol, and ellagic acid; the latter of which urolithin is obtained. Other fruits such as cherries, strawberries, grapes, and apples also contain catechin, quercetin, and ellagic acid. Additionally, vegetables such as onions, cabbage, cauliflower, and capers contain important levels of quercetin. Walnuts and peanuts present both resveratrol and ellagic acid. All these compounds make up important components of the Mediterranean diet, and therefore, in this paper, we review the influence of these four polyphenols on overall health, mainly focusing on their effects on endothelial function and cardiovascular health.

### 1.4. Aging

Out of the theories of aging, those related to endothelial dysfunction include the theory of free radicals, mitochondrial dysfunction, the role of the insulin pathway, telomeric attrition, and senescence [14].

The idea of cellular senescence contributing to the aging process is supported by the finding that senescent cells halt normal function, irreversibly cease dividing, and accumulate in aging organisms, thereby causing dysfunction through inflammation and atherosclerotic plaques [15]. Endothelial cell senescence is induced by multiple factors, such as oxidative stress, vascular inflammation, and dysregulation of the cell cycle, which causes structural and functional changes, such as increased permeability of the endothelium, alterations in mitochondrial biogenesis, impairment of vascular repair, and angiogenesis [16]. Another hallmark of aging is telomere attrition. In human aortic endothelial cells, the inhibition of telomere function induces the expression of ICAM-1 and reduces eNOS expression, indicating that endothelial function is impaired in aging and cellular senescence [14, 17]. The four polyphenols outlined in this review have been shown to have mediating effects on both ICAM-1 and eNOS expressions, indicating that they might have the ability to slow the shortening of telomeres caused by aging.

The IIS pathway is the most conserved aging-controlling pathway in evolution, and among its multiple targets are the FOXO family of transcription factors and the mTOR complexes. The two nutrient sensors, AMPK and sirtuins, act in the opposite direction to IIS and mTOR, and their upregulation favor healthy aging [14]. Mitochondrial function becomes perturbed by aging-associated mtDNA mutations, and defective mitochondrial function generates ROS that, below a certain threshold, induce survival signals to restore cellular homeostasis, but at higher or continued levels contribute to aging [14]. There is compelling evidence that polyphenols such as catechin, quercetin, and resveratrol inhibit mitochondrial ATP and thereby increase AMP levels and activation of AMPK, which promotes mitochondrial biogenesis and healthy aging [18]. Furthermore, resveratrol protects from metabolic damage and improve mitochondrial respiration in a PGC-1*α*-dependent manner [19].

As we will see, several polyphenols improve endothelial function by acting on mediators that are also described in the hallmarks of aging, such as telomerase attrition and mitochondrial function.

### 1.5. Metabolism

Recent studies have shown cell communication pathways linked to diabetes mellitus and obesity and how they may affect inflammatory and vascular health [20]. Therefore, a model to monitor endothelial dysfunction is the induction of diabetes, which is widely associated with cardiovascular diseases. Another measurable factor is obesity, which increases the risk of insulin resistance, disrupts vascular homeostasis, and contributes to endothelial vasodilator dysfunction and subsequent hypertension [21]. There is compelling evidence that the polyphenols outlined in this review can aid in weight loss and thereby decrease the risk of vascular metabolic diseases. Thus, consumption of polyphenols to maintain a healthy weight could decrease the risk of, and potentially even prevent, endothelial dysfunction before symptoms arise. Moreover, evidence shows that resveratrol increases lipolysis not only in obese animals but also in healthy, aged mice [22].

Based on this evidence, we propose in this review to study the role of quercetin, catechin, resveratrol, and urolithin on the endothelial dysfunction and related cardiovascular diseases.

## 2. Quercetin

Quercetin is a polyphenol belonging to the flavonoid family with anti-inflammatory, antioxidant and possible senolytic properties, among others [23–25]. It can be found in many plant foods, such as onions, apples, and blueberries, as well as in tea and red wine. Quercetin is not only beneficial in diabetic or inflammatory conditions; it also induces reduction of fatty acid and triacylglycerol synthesis and inhibits LDL oxidation in hepatocytes of healthy rats, thereby promoting cardiovascular protective functions [26, 27].

In regard to the mechanisms of the control of endothelial function, Akt, a downstream protein effector of PI3K, plays an important role in improving vasodilatory actions of insulin in human endothelium, thereby providing protective effects [28]. In this context, quercetin increases Akt phosphorylation and subsequently eNOS phosphorylation and NO production in response to insulin through inflammatory pathways in diabetic rats [29]. Additionally, quercetin at concentrations of 50 mg/kg exerts vasoprotective effects against endothelial dysfunction induced by endoplasmic reticulum stress. These effects are mediated by increased expression of VEGF in streptozotozine-induced diabetic rats [30], which is a model to study endothelial function.

ICAM-1 plays an essential role in inflammatory responses. The TNF-*α*-induced expression of ICAM-1 was shown to be reduced in an *in vitro* study with human endothelial cells when treated with 10–50 *μ*M quercetin [31]. Another study showed a significant inhibitory effect of 50 *μ*M treatment of quercetin on VCAM-1, ICAM-1, and E-selectin expressions. Inhibitory effects on COX-2 and iNOS protein levels were also found when treated with concentrations of 5–50 *μ*M [32]. Quercetin has also been shown to activate the expression of the protein HIF-1*α*, a key transcription factor in the induction of the VEGF gene, as well as in regulating glucose and hypoxia homeostasis, potentially through its antioxidative properties, when treated with concentrations of 10 mM [33].

Furthermore, quercetin has been shown to reduce body weight gain in high-fat diet-fed mice by lowering serum lipid levels and hepatic lipid accumulation. This is mediated through the downregulation of cytochrome p450 2b genes, which intervene in the synthesis of steroids, such as cholesterol, and are therefore important in hepatic lipid homeostasis [34, 35]. However, in this study, quercetin was administered in 0.3% *w*/*w* dosages to rats in order to examine quercetin's effect in hepatic lipid metabolism [34]. This dosage indicates that a 70 kg individual would have to consume approximately 200 grams of quercetin supplements to achieve the same results.

Even though some studies have concluded that quercetin might not be effective as a senolytic in endothelial cells, a clinical trial conducted in 2019 showed that quercetin is effective in decreasing senescent cells [25, 36]. However, as this is the first direct evidence that senolytics are effective in humans, further research is necessary to confirm the effects of quercetin in regard to senescence.

These results show that quercetin exerts multiple health-promoting effects through an increase in vascular endothelial mediators, as well as possible weight loss, anti-aging, and senolytic benefits.

## 3. Catechin

Catechin is a flavanol present in many plants and is especially abundant in berries, cacao, and green tea [37]. A positive correlation between green tea and cardiovascular health has been found due to catechin's antioxidant, anti-inflammatory, antihypertensive, and antihyperlipidemic properties [38].

Catechin supplementation has been shown to significantly increase flow-mediated dilation and significantly reduce pulse wave velocity, diastolic blood pressure, and augmentation index, which are parameters for cardiovascular health in humans. This has been shown in several studies performed in both young and elderly healthy individuals as well as in individuals with cardiovascular disease risk through the administration of pure catechin supplements in quantities between 20 and 300 mg/day and extracts from foods rich in catechins, such as apples, grapes, and cacao in quantities estimated between 7.5 and 146 mg/day in a time period ranging between 2 and 12 weeks [39, 40].

Furthermore, one study showed that even in exceptionally low quantities, catechin exerted protective effects. The intake of red grape polyphenol extract containing only 2.72 mg catechin was shown to improve endothelial function in patients with cardiovascular disease. In this study, 30 male patients with coronary heart disease were randomly assigned into two groups where one received polyphenol extract dissolved in water, and a placebo group received water without supplementation. The group consuming polyphenol extract showed significant increased flow-mediated dilatation compared to the placebo group, with a peak at 60 min after intake [41].

In addition to the cardiovascular and endothelial protective effects of catechin, it has also been shown to reduce body fat. Interventional studies of 3 daily cups of green tea after meals showed an average metabolic boost of about one hundred calories a day by promoting lipidic oxidation [42]. A possible explanation for why green tea consumption prolongs diet-induced thermogenesis is catechin's ability to inhibit catechol-O-methyltransferase, the enzyme that degrades norepinephrine [43]. However, in other studies, catechol-O-methyltransferase was not shown to be suppressed after consumption of catechin [44]. Therefore, a more plausible explanation for its thermogenesis-promoting effects is through brown adipose tissue signaling through evoking white adipocyte beiging and suppression of adipogenesis [45].

Moreover, daily intake of catechin-comprised red wine extract has shown to have a beneficial effect on aortic expression of endothelial dysfunction biomarkers. These include VCAM-1, ICAM-1, E-selectin, and LOX-1 as well as proinflammatory cytokines such as TNF-*α* and IL-6 in hyperhomocysteinemic mice [46]. Catechin also improves redox imbalance and mitochondrial dysfunction, a prominent feature of cardiovascular disease, by stimulating phosphorylation of AMPK and ACC, inhibiting the key enzymes of de novo lipogenesis, and blocking the TNF-*α*-induced insulin signaling pathway [45, 47]. Furthermore, it significantly induces PGC-1*α*, which in turn stimulates mitochondrial proliferation [48].

These results suggest that consumption of catechin-rich food could be useful in combatting endothelial dysfunction both through direct signaling pathways, as well as indirectly through reducing risk factors of obesity-related metabolic diseases, such as diabetes and cardiovascular disease.

## 4. Resveratrol

Resveratrol is one of the most studied plant secondary metabolites [49]. Experimental studies have shown benefits of resveratrol on the cardiovascular system through blockage of low-density lipoprotein, cholesterol peroxidation, reduction of ROS, and prevention of platelet aggregation by promoting vasodilation through NO synthesis and inhibition of ET-1 [50–53].

In a randomized, double-blind, placebo-controlled study on 24 hypertensive patients with endothelial dysfunction, a single dose of 300 mg of resveratrol supplement was shown to increase branchial flow-mediated dilation response and thereby improve endothelial function [54]. In a similar study, resveratrol improved flow-mediated dilation of the brachial artery of 19 overweight men and women with hypertension in a dose-dependent manner, with an increase from 4.1 ± 0.8% measured in the placebo group to 7.7 ± 1.5% after the intake of 270 mg resveratrol 3 times weekly [55].

Studies *in vitro* further confirm the beneficial effects of resveratrol on endothelial metabolism and inflammation. In a study with TNF-*α*-activated endothelial cells, the polyphenol intervention of 5-20 *μ*M significantly reduced the levels of VEGF, ROS, and proinflammatory mediator such as IL-8 and ICAM-1 [53].

PPAR*δ*, which has been shown to have increased activity in endothelial cells after resveratrol consumption in dosages of 20 mg/kg/day over a two-week span, contributes to the beneficial effect of SIRT1 to ameliorate endothelial dysfunction in diabetic and obese mice [56].

Additionally, PPAR*δ* activation by synthetic ligands protects against obesity, decreases triglyceride levels, increases HDL-cholesterol, and improves insulin sensitivity and could therefore also be helpful in regard to weight loss [57, 58]. 150 mg/day of resveratrol intake in 11 obese individuals over 30 days in a randomized double-blind crossover study resulted in metabolic changes mimicking the effects of calorie restriction [59]. These changes included reduced systolic blood pressure, adipose tissue lipolysis, plasma fatty acid, and glycerol levels and increased expression of AMPK, SIRT1, and PGC-1*α* protein levels.

Increased expression of AMPK*α* by resveratrol treatment has also been found to be effective in elderly mice. In a study where 12 healthy, elderly mice were supplemented with 2.3 *μ*g/kg/day of resveratrol for two consecutive days, fatty acid mobilization and degradation increased, and their synthesis was inhibited [22].

From this, we can conclude that resveratrol consumption can aid in weight loss and aging, prevent metabolic diseases before they arise, and improve endothelial metabolism in those with inflammatory states and cardiovascular-related diseases.

## 5. Urolithin

Urolithins are the major metabolites of dietary ellagic acid, found in the form of ellagitannin in foods such as walnuts, strawberries, and pomegranate. The three most abundant bioavailable ellagitannin metabolites are urolithins A, B, and C, which are transformed by the gut bacteria *Gordonibacter urolithinfaciens* and *Gordonibacter pamelaeae* [60].

Numerous studies indicate that apart from their anti-inflammatory effects, urolithin exhibits antiatherosclerotic and anticarcinogenic effects, which support their potential preventive effect against cardiovascular diseases [61, 62]. One of the mechanisms by which quercetin acts is through inhibiting iNOS production and decreasing the expression of the proinflammatory cytokines IL-1*β*, TNF-*α*, and IL-6 [63, 64].

A double-blind randomized controlled crossover human intervention trial with 10 healthy males confirmed that ellagitannins acutely improve endothelial function through increasing flow-mediated dilation after the consumption of red raspberries in 200 and 400 g quantities when compared to a placebo group [65]. In another study where human artery endothelial cells were incubated with 50 *μ*g/mL ox-LDL, urolithin A improved the productions of NO and eNOS in a dose-dependent manner [66]. Additionally, it significantly suppressed expressions of TNF-*α*, IL-6, ICAM-1, MCP-1, and ET-1 and increased PPAR-*γ* mRNA levels [66]. The beneficial effects attributed to the consumption of ellagitannin-containing foods against cardiovascular diseases were confirmed in another study where human aortic endothelial cells were exposed to TNF-*α* and treatment with Uro-A glucuronide inhibited monocyte adhesion and endothelial cell migration and decreased the expression of CCL-2 and IL-8 [61].

*In vivo* animal studies have shown that treatment with ellagic acid extracted from raspberry seed flour in concentrations estimated to 0.03% of the total diet lowers the elevated triglyceride and LDL cholesterol levels and oxidative stress provoked by a high-fat high-sucrose diet [67]. Another study showed that treatment of 7.5 and 15 mg/kg for 10 days reduced LDL, free fatty acids, triglyceride levels, and lipid peroxidation, while exerting cardiac protective effects by reducing ventricular hypertrophy and arrhythmia in myocardial infarction-induced rats [68].

In regard to obesity, urolithin A has been shown to reduce triglyceride accumulation, lipogenesis and gene expression related to adipocyte formation such as adiponectin, GLUT4, and FABP4 in 3T3-L1 adipocytes [69].

These results suggest that urolithin A has protective benefits against inflammation states and metabolic-related diseases, such as diabetes and obesity, which in turn are risk factors for cardiovascular disease and endothelial dysfunction.

The following table presents the mechanisms through which each of the four outlined polyphenols exert their protective effects as found through studies on humans, animals, and *in vitro* (Table 1).

## 6. Discussion

The exerted effects of catechin, quercetin, urolithin, and resveratrol on endothelial function are beneficial not only for patients with cardiovascular disease, diabetes, or endothelial dysfunction but also for all healthy individuals as it can improve the effects of aging on the endothelia and aid in weight loss, thereby further improving health status while lowering the risk of several diseases, including those caused by endothelial dysfunction.

In 2016, an article was published outlining the relation between a lower incidence of cardiovascular disease and the ingestion of antioxidants performed on 800 individuals in Holland. This effect was attributed to the consumption of flavonoids present in the diet, such as in apples, onion, and tea. Although the exact compounds responsible for this effect were not determined, the authors described quercetin as one of them, as it was the compound with the highest antioxidant effect, due to its molecular structure [23]. Previous *in vitro* studies performed with human endothelial cells show that quercetin exerts not only antioxidant effects but also anti-inflammatory and vasodilator effects though downregulation of ACE [24]. Additionally, in these same cells, the anti-inflammatory effects of quercetin mediated by a decrease in I-CAM, NF-*κ*B, and AP-1 have been discovered [31, 32]. Furthermore, studies *in vitro* performed on rat liver cells have shown that quercetin diminishes the synthesis of fatty acids and triglycerides [26]. These data could explain the decrease in cardiovascular disease risk in the studies previously cited. However, the concentrations used in the studies *in vitro* are of micromolar magnitudes, which cannot be reached through diet and can only be achieved through supplementations. Toxicity studies have been performed in humans, where it has been stated that concentrations up to 1 g/day of quercetin does not pose any harm. Should one surpass this concentration however, quercetin could become prooxidant and thereby exert genotoxic effects [70].

The studies on the benefits of catechin also showed enhancements of the cardiovascular system through antioxidant, anti-inflammatory, antiproliferative, antithrombogenic, and antihyperlipidemic mechanisms [38]. The studies performed on humans with catechin and epigallocatechin supplements vary in dosages between 20 and 300 mg/day, with variations in duration between 2 and 6 weeks, where increased flow-mediated dilation was found. These findings, along with those previously described, confirm that the consumption of catechin favors cardiovascular health [39]. Additionally, dietary intake of catechin through tea, grapes, or cacao has been proven to favor the increase in flow-mediated dilation in concentrations estimated to 146 mg from cacao and 7.5 mg from grapes. However, as these aliments contain a multitude of polyphenols, the suggested mechanisms on the cardiovascular system could be affected by other compounds. It is important to note that the outlined benefits were seen both in patients with cardiovascular diseases and obese individuals, as well as in young and elderly individuals [39–41, 44]. Concerning dosage, there have been no reported findings of toxicity from catechin, although it is possible that it could follow the same guidelines as quercetin.

Resveratrol is one of the most studied polyphenols. It can be absorbed quickly due to its lipophilic characteristics and even more so when ingested with foods rich in fats or with alcohol such as red wine. However, it is quickly metabolized in the liver and therefore, its distribution is highly variable [71]. As shown in this review, resveratrol clearly improves lipidic profiles and endothelial smooth muscle relaxations in hypertense and obese patients [54, 55]. In animal studies, it has been shown how this polyphenol increases lipid catabolism in skeletal muscle, which could possibly explain the changes in lipidic profiles in humans [71]. Moreover, studies have shown the protective effects of resveratrol in ischemic reperfusion-induced rats, mediated by autophagia and the mTOR signaling pathway [51]. In endothelial cells, resveratrol has been shown to decrease TNF-*α* levels and thereby prevent inflammation [53], as well as protect against lipidic peroxidation in human monocytes [52]. Based on these findings, we can conclude that the authors of these studies agree that resveratrol exerts beneficial effects on the cardiovascular system.

Human studies relating urolithin to endothelial function are scarce. However, in one study performed on healthy individuals where 200-400 g of raspberry drink was administered, the presence of ellagitannins, the precursors of urolithin, was found present in the blood, in nanomolar magnitudes, with an augmented capacity for flow-mediated dilation [65], results similar to those found in quercetin. It is important to note that the exerted effects were likely not exclusively caused by urolithin, as the drink was rich in polyphenolic compounds. Interestingly, in the study performed on myocardial infarction-induced rats, although urolithin was found to exert protective benefits on the cardiovascular system, there was no significant difference between the groups administered 7.5 and 15 mg/day, suggesting a saturation of the protective effects at the lower dosage. Moreover, in the control groups, there was no significant difference found in the lipidic profile in the nonsupplemented healthy animals and the healthy animals administered urolithin, suggesting that these dosages are not beneficial in healthy animals [68]. Another study performed on animals administered a diet high in fats and sugar supplemented with 100 mg of raspberry extract showed an improvement in lipidic profile and parameters of inflammation and oxidative stress [67]. It would be interesting to confirm these results with their concrete mechanisms and signaling pathways. The *in vitro* studies outlined in this review confirm some of these parameters. It has been shown how 5-15 *μ*M of urolithin exerts anti-inflammatory effects on endothelial cells [61]. Additionally, with these same concentrations, an anti-inflammatory pathway through activation of macrophages with lipopolysaccharides has been confirmed [63, 64]. Other action mechanisms suggest a signaling pathway through microRNA-27 and ERK/PPAR-*γ* in endothelial dysfunction caused by oxidized cholesterol treated with 5 *μ*M [66]. It is important to note that the concentrations of urolithin in the previously cited human study are administered in nanomolar magnitudes and it could therefore be interesting to perform studies *in vitro* to verify if the effects of these nutritionally relevant concentrations are beneficial.

The conditions in which polyphenols are consumed should also be taken into consideration; as for both quercetin and resveratrol, marked differences in functional effects were observed dependent on standard or high-fat dietary backgrounds [72]. As mentioned, it is important to note that the majority of the studies mentioned in this review were performed with polyphenols in supplemental quantities, rather than from natural plant sources, making the dosage recommendations for humans difficult to determine. Some of the amounts used in these trials may not be nutritionally relevant, and although no toxicity has been found in the articles outlined in this review when administered for up to 3 months, the extrapolation of the concentrations administered in some animal studies could prove to cause acute toxicity in humans. Further studies should be conducted with these polyphenols in quantities possible to achieve through diet. It would also be interesting to study these four polyphenols simultaneously, to see if they act upon common signaling pathways, if they have synergic effects, or if the beneficial effects could be increased when working together.

## 7. Conclusion

**Figure 2:**
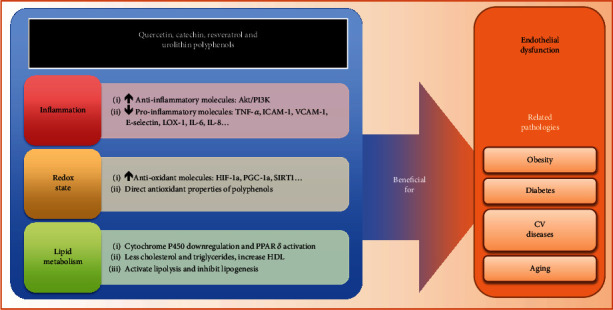
The effects of polyphenols in the Mediterranean diet on pathologies related to endothelial dysfunction.

Thus, we can conclude that the polyphenols quercetin, catechin, resveratrol, and urolithin could be an effective tool, as they could serve as pharmacologic targets in treatments for diseases related to the cardiovascular system. These polyphenols can be used in combination with pharmacological treatments such as anti-inflammatory, anticoagulant, and antihypertensive drugs, to improve the damage caused by endothelial dysfunction (Figure 2). Additionally, they can exert protective effects in healthy individuals to reduce the risk of cardiovascular diseases.

## Figures and Tables

**Table 1:** Outlined effects of quercetin, catechin, resveratrol, and urolithin studied *in vitro*, in animals, and in humans.

Molecule	Study type	Mechanisms	Refs.
Quercetin	*In vitro*	Reduces TNF-*α*-induced expression of ICAM-1 Inhibits VCAM-1 and E-selectin expression, as well as COX-2 and iNOS Activates the expression of HIF-1*α* and regulates glucose and hypoxia homeostasis	[31] [32] [33]
	Animal	Induces reduction of fatty acid and triacylglycerol synthesis and inhibits LDL oxidation Increases Akt phosphorylation, eNOS phosphorylation, and NO production Increases expression of VEGF Downregulates cytochrome p450 2b genes, steroid, and cholesterol synthesis	[26, 27] [28, 29] [30] [34, 35]
	Human	Reduces cellular senescence	[25]
Catechin	*In vitro*	Improves redox imbalance and mitochondrial dysfunction by AMPK and ACC phosphorylation Inhibits TNF-*α*-induced insulin signaling pathway and induces PGC-1*α* expression	[47] [48]
	Animal	Exerts beneficial effects on aortic expression of endothelial dysfunction biomarkers (VCAM-1, ICAM-1, E-selectin, LOX-1, TNF-*α*, and IL-6)	[46]
	Human	Increases flow-mediated dilation, reduces pulse wave velocity and diastolic blood pressure Increases thermogenesis, reduces adipogenesis, and promotes fat oxidation	[39–41] [42, 43]
Resveratrol	*In vitro*	Reduces cholesterol peroxidation, reactive oxygen species levels, and platelet aggregation by promoting vasodilation through NO synthesis and inhibition of endothelin-1 Lowers proinflammatory mediators IL-8 and ICAM-1 in TNF-*α-*activated endothelial cells	[50–52] [53]
	Animal	Ameliorates endothelial dysfunction through PPAR*δ* and SIRT1 activation Increases expression of AMPK*α*, activates fatty acid mobilization and degradation, and inhibits fatty acid synthesis	[56] [22]
	Human	Increases branchial flow-mediated dilation response Rises the expression of AMPK, SIRT1, and PGC-1*α* protein levels	[54, 55] [59]
Urolithin	*In vitro*	Inhibits monocyte adhesion, endothelial cell migration, and CCL-2 and IL-8 expressions Inhibits iNOS and decreases proinflammatory cytokines expression (IL-1*β*, TNF-*α*, and IL-6) Improves NO production and eNOS activity in a dose-dependent manner and suppresses the expression of TNF-*α*, IL-6, ICAM-1, MCP-1, and endothelin 1 and increases PPAR-*γ* mRNA levels	[61] [63, 64] [66]
	Animal	Reduces LDL cholesterol, triglycerides, free fatty acids, and oxidative stress Reduces ventricular hypertrophy and arrhythmia	[67, 68] [68]
	Human	Improves endothelial function through improvements in flow-mediated dilation	[65]

## Abbreviations

ACC: Acetyl-CoA carboxylase
AMPK: 5′AMP-activated protein kinase
ANG II: Angiotensin II
CAMs: Cell adhesion molecules
ENOS: Endothelial nitric oxide synthase
EDHF: Endothelium-derived hyperpolarizing factor
EGF: Epidermal growth factor
E-selectin: Endothelial-leukocyte adhesion molecule 1
ET-1: Endothelin-1
FABP4: Fatty acid binding protein 4
FGF: Fibroblast growth factor
GLUT4: Glucose transporter type 4
H2S: Hydrogen sulfide
HSPG: Heparan sulfate proteoglycan
ICAM-1: Intercellular adhesion molecule-1
IIS: The insulin/IGF-1 signaling
LDL: Low-density lipoprotein
LOX-1: Oxidized low-density lipoprotein receptor 1
MCP-1: Monocyte chemotactic protein 1
NO: Nitric oxide
PAI-1: Plasminogen activator inhibitor-1
PDGF: Platelet-derived growth factor
PGC-1*α*: Peroxisome proliferator-activated receptor gamma coactivator 1-alpha
PGI2: Prostaglandin I2
PGH2: Prostaglandin H2
PPAR*δ*: Peroxisome proliferator-activated receptor gamma
ROS: Reactive oxygen species
SIIR: Steroid-induced insulin resistant
SIRT1: Sirtuin 1
TGF*-β*: Transforming growth factor *β*
THP-1: Human acute monocytic leukemia cell line
TNF-*α*: Tumor necrosis factor*-α*
tPA: Tissue plasminogen activator
TXA2: Thromboxane A2
VCAM-1: Vascular cell adhesion molecule 1
VEGF: Vascular endothelial growth factor
vWF: von Willebrand factor.
